# A monoclinic polymorph of KY(PO_3_)_4_

**DOI:** 10.1107/S1600536808013925

**Published:** 2008-05-17

**Authors:** Karima Horchani-Naifer, Anis Jouini, Mokhtar Férid

**Affiliations:** a Unité de Recherches de Matériaux de Terres Rares, Centre National de Recherches en Sciences des Matériaux, BP 95 Hammam-Lif 2050, Tunisia

## Abstract

The title compound, potassium yttrium polyphosphate, KY(PO_3_)_4_, was synthesized using the flux method. The atomic arrangement consists of an infinite long-chain polyphosphate organization. Chains, with a period of four PO_4_ tetra­hedra, run along the *a*-axis direction. Two other polymorphs of this phosphate are known, in space groups *P*21/*n* and *C*2/*c*.

## Related literature

For related structures, see: Durif (1995 ▶); Hamady *et al.* (1995 ▶); Hong *et al.* (1975 ▶); Malinowski (1990 ▶); Malinowski *et al.* (1988 ▶); Palkina *et al.* (1977 ▶). For earlier work on KY(PO_3_)_4_, see: Jouini *et al.* (2003 ▶). For related literature, see: Sun *et al.* (2004 ▶).

## Experimental

### 

#### Crystal data

KY(PO_3_)_4_
*M* *_r_* = 443.89
Monoclinic, 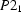

*a* = 7.2244 (3) Å
*b* = 8.2825 (3) Å
*c* = 7.854 (4) Å
β = 91.735 (3)°
*V* = 469.7 (2) Å^3^
*Z* = 2
Mo *K*α radiation
μ = 7.40 mm^−1^
*T* = 298 (2) K
0.16 × 0.14 × 0.13 mm

#### Data collection

Enraf–Nonius CAD-4 diffractometer
Absorption correction: multi-scan (*SADABS*; Sheldrick, 1996 ▶) *T*_min_ = 0.321, *T*_max_ = 0.376
3651 measured reflections
2011 independent reflections
1904 reflections with *I* > 2σ(*I*)
*R*_int_ = 0.081
2 standard reflections every 150 reflections intensity decay: 2%

#### Refinement

*R*[*F* ^2^ > 2σ(*F* ^2^)] = 0.048
*wR*(*F* ^2^) = 0.138
*S* = 1.13
2011 reflections
165 parameters
1 restraint
Δρ_max_ = 1.19 e Å^−3^
Δρ_min_ = −2.67 e Å^−3^
Absolute structure: Flack (1983 ▶), with 867 Friedel pairs
Flack parameter: 0.002 (9)

### 

Data collection: *CAD-4 EXPRESS* (Duisenberg, 1992 ▶; Macíček & Yordanov, 1992 ▶); cell refinement: *CAD-4 EXPRESS*; data reduction: *XCAD4* (Harms & Wocadlo, 1995 ▶); program(s) used to solve structure: *SHELXS97* (Sheldrick, 2008 ▶); program(s) used to refine structure: *SHELXL97* (Sheldrick, 2008 ▶); molecular graphics: *DIAMOND* (Brandenburg, 2001 ▶); software used to prepare material for publication: *SHELXL97*.

## Supplementary Material

Crystal structure: contains datablocks global, I. DOI: 10.1107/S1600536808013925/br2073sup1.cif

Structure factors: contains datablocks I. DOI: 10.1107/S1600536808013925/br2073Isup2.hkl

Additional supplementary materials:  crystallographic information; 3D view; checkCIF report

## Figures and Tables

**Fig. 1.**
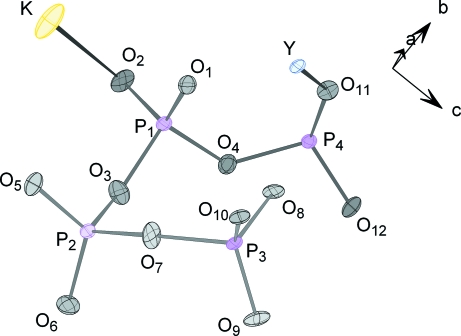
: Projection of KY(PO3)4 with anisotropic displacement parameters drawn at the 50% probability level.

**Fig. 2.**
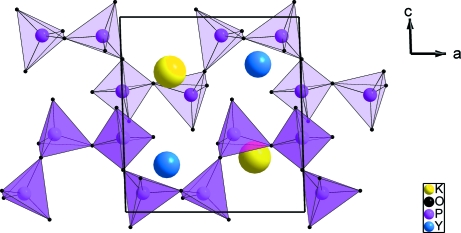
: The structural arrangement of KY(PO3)4 along the b axis.

**Fig. 3.**
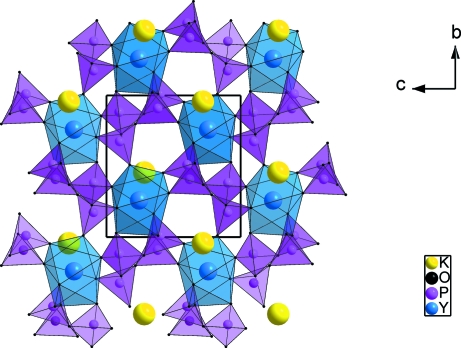
: Projection of KY(PO3)4 along the a axis, showing isolated YO8 polyhedra joined to (PO3)n chains.

## supplementary crystallographic information

### Comment

Yttrium condensed phosphates have been considered as a crystal hosts for optical materials when doped with lanthanides, due to there high-temperature chemical stability, high yield intrinsic fluorescence and minimal trapping of excitation, rendering them attractive materials for investigations of the energy transfer phenomena and fluorescence quenching(Malinowski, 1990; Malinowski *et al.*,  1988). The literature dealing with these compounds was rather confusing for some time, between cyclic or chain condensed phosphates, but it is currently well established that the *M*^I^Y(PO_3_)_4_ compounds are polyphosphates with infinite chain and *M*^I^YP_4_O_12_ are cyclotetraphosphates (with *M*^I^= monovalent cation) (Durif, 1995). In our laboratory we have synthesized the potassium and yttrium polyphosphates to establish the solid–liquid equilibrium diagram of the KPO_3_–Y(PO_3_)_3_ system (Jouini *et al.*,  2003). Three allotropic phases with the space groups *P*2_1_, *P*2_1_/*n* and *C*2/*c* were isolated and characterized. The three monoclinic allotropes are: i) KY(PO_3_)_4_ polyphosphate with the *P*2_1_ space group, isostructural with KNd(PO_3_)_4_ (Hong, 1975). ii) KY(PO_3_)_4_ polyphosphate belongs to *P*2_1_/*n* space group, and is isostructural with TlNd(PO_3_)_4_ (Palkina *et al.*,  1977). In these two forms the phosphate anion has a chain structure. iii) The third allotropic form is KYP_4_O_12_ which crystallizes in the *C*2/*c* space group, only this structure was investigated (Hamady, 1995). This paper is devoted to the crystal structure of the first polymorph KY(PO_3_)_4_ (*P*2_1_). The atomic arrangement of this srtucture is characterized by a three-dimensional framework built of (PO_3_)_n_ chains that are formed by corner-sharing of PO_4_ tetrahedra (Figs 1,2). The chains run along the *a* axis , with four PO_4_ tetrahedra in a repeating unit. KY(PO_3_)_4_ is isostructural with KNd(PO_3_)_4_ , but not with CsLa(PO_3_)_4_ (Sun *et al.*,  2004) although they belong to the same space group, *P*2_1_. In the latter, the infinite screw (PO_3_)_n_ chains are repeated after every eighth PO_4_ group along the b axis. The chains (two per unit cell) are joined to each other by YO_8_ polyhedra (Fig 3.), no O atom is shared with the adjacent YO_8_ polyhedra. The K atoms are in an eightfold coordination.

### Experimental

Single crystal of KY(PO_3_)_4_ was prepared by flux method. Homogeneous solution of potassium carbonate K_2_CO_3_ (6 g) and yttrium oxide Y_2_O_3_ (0.5 g) containing a large excess of orthophosphoric acid H_3_PO_4_ (16 ml, 85% concentration) was heated in a vitreous carbon crucible at 473 K for 1 day. Then the temperature of the furnace was slowly raised to the predermined temperature in the range of 573–623 K for 7 days. Crystals were separated from the excess phosphoric acid by washing the product in boiling water.

### Refinement

The highest peak and the deepest hole are located 0.09Å and 0.85 Å from Y.

### Crystal data

**Table:**

KY(PO_3_)_4_	*F*_000_ = 428
*M**_r_* = 443.89	*D*_x_ = 3.138 Mg m^−^^3^
Monoclinic, *P*2_1_	Melting point: 760 K
Hall symbol: P 2yb	Mo *K*α radiation λ = 0.71073 Å
*a* = 7.2244 (3) Å	Cell parameters from 25 reflections
*b* = 8.2825 (3) Å	θ = 2.6–27.5º
*c* = 7.854 (4) Å	µ = 7.40 mm^−^^1^
β = 91.735 (3)º	*T* = 298 (2) K
*V* = 469.7 (2) Å^3^	Prism, colourless
*Z* = 2	0.16 × 0.14 × 0.13 mm

### Data collection

**Table:**

Enraf–Nonius CAD-4 diffractometer	*R*_int_ = 0.081
Radiation source: fine-focus sealed tube	θ_max_ = 27.5º
Monochromator: graphite	θ_min_ = 2.6º
*T* = 298(2) K	*h* = −7→9
ω/2θ scans	*k* = −8→10
Absorption correction: multi-scan(SADABS; Sheldrick, 1996)	*l* = −8→10
*T*_min_ = 0.321, *T*_max_ = 0.376	2 standard reflections
3651 measured reflections	every 150 reflections
2011 independent reflections	intensity decay: 2%
1904 reflections with *I* > 2σ(*I*)	

### Refinement

**Table:**

Refinement on *F*^2^	Secondary atom site location: difference Fourier map
Least-squares matrix: full	*w* = 1/[σ^2^(*F*_o_^2^) + (0.0908*P*)^2^] where *P* = (*F*_o_^2^ + 2*F*_c_^2^)/3
*R*[*F*^2^ > 2σ(*F*^2^)] = 0.048	(Δ/σ)_max_ < 0.001
*wR*(*F*^2^) = 0.138	Δρ_max_ = 1.19 e Å^−^^3^
*S* = 1.13	Δρ_min_ = −2.67 e Å^−^^3^
2011 reflections	Extinction correction: SHELXL97 (Sheldrick, 2008), Fc^*^=kFc[1+0.001xFc^2^λ^3^/sin(2θ)]^-1/4^
165 parameters	Extinction coefficient: 0.065 (7)
1 restraint	Absolute structure: Flack (1983), with 867 Friedel pairs
Primary atom site location: structure-invariant direct methods	Flack parameter: 0.002 (9)

### Special details

**Table:**

Geometry. All esds (except the esd in the dihedral angle between two l.s. planes) are estimated using the full covariance matrix. The cell esds are taken into account individually in the estimation of esds in distances, angles and torsion angles; correlations between esds in cell parameters are only used when they are defined by crystal symmetry. An approximate (isotropic) treatment of cell esds is used for estimating esds involving l.s. planes.
Refinement. Refinement of F^2^ against ALL reflections. The weighted R-factor wR and goodness of fit S are based on F^2^, conventional R-factors R are based on F, with F set to zero for negative F^2^. The threshold expression of F^2^ > 2sigma(F^2^) is used only for calculating R-factors(gt) etc. and is not relevant to the choice of reflections for refinement. R-factors based on F^2^ are statistically about twice as large as those based on F, and R- factors based on ALL data will be even larger.

### Fractional atomic coordinates and isotropic or equivalent isotropic displacement parameters (Å^2^)

**Table:**

	*x*	*y*	*z*	*U*_iso_*/*U*_eq_	
Y	0.23704 (8)	0.75897 (9)	0.24245 (8)	0.0086 (3)	
K	0.2703 (3)	0.4566 (3)	−0.2812 (3)	0.0303 (6)	
P1	0.4367 (3)	0.3830 (2)	0.0947 (3)	0.0091 (4)	
P2	0.0994 (3)	0.1755 (2)	0.0978 (2)	0.0086 (4)	
P3	−0.0018 (3)	0.4085 (2)	0.3809 (2)	0.0088 (4)	
P4	0.6161 (3)	0.5114 (2)	0.3992 (2)	0.0084 (4)	
O1	0.3212 (8)	0.5292 (7)	0.0721 (7)	0.0140 (12)	
O2	0.5736 (8)	0.3558 (8)	−0.0387 (8)	0.0184 (13)	
O3	0.3126 (7)	0.2261 (7)	0.1093 (8)	0.0169 (13)	
O4	0.5360 (8)	0.3706 (7)	0.2789 (7)	0.0139 (12)	
O5	0.0239 (8)	0.2062 (7)	−0.0776 (7)	0.0150 (12)	
O6	0.0880 (8)	0.0105 (7)	0.1732 (7)	0.0163 (12)	
O7	−0.0076 (8)	0.2991 (7)	0.2159 (8)	0.0189 (13)	
O8	0.1660 (7)	0.5100 (7)	0.3846 (7)	0.0124 (11)	
O9	−0.0337 (8)	0.3109 (7)	0.5348 (8)	0.0167 (12)	
O10	−0.1755 (8)	0.5225 (7)	0.3407 (7)	0.0121 (11)	
O11	0.5258 (8)	0.6648 (7)	0.3514 (7)	0.0138 (11)	
O12	0.6118 (7)	0.4535 (7)	0.5773 (7)	0.0121 (11)	

### Atomic displacement parameters (Å^2^)

**Table:**

	*U*^11^	*U*^22^	*U*^33^	*U*^12^	*U*^13^	*U*^23^
Y	0.0092 (4)	0.0103 (4)	0.0067 (4)	−0.0004 (3)	0.0048 (2)	−0.0001 (2)
K	0.0180 (10)	0.0601 (16)	0.0129 (8)	0.0108 (9)	0.0031 (7)	−0.0019 (10)
P1	0.0100 (9)	0.0098 (10)	0.0080 (9)	−0.0003 (7)	0.0044 (7)	−0.0004 (6)
P2	0.0084 (9)	0.0095 (9)	0.0082 (9)	−0.0008 (6)	0.0034 (7)	−0.0004 (7)
P3	0.0090 (9)	0.0107 (10)	0.0070 (9)	−0.0009 (7)	0.0055 (7)	0.0003 (7)
P4	0.0086 (9)	0.0088 (9)	0.0081 (8)	0.0003 (6)	0.0032 (7)	0.0001 (6)
O1	0.018 (3)	0.012 (3)	0.012 (3)	0.001 (2)	0.003 (2)	−0.001 (2)
O2	0.021 (3)	0.021 (3)	0.014 (3)	0.007 (2)	0.011 (2)	−0.001 (2)
O3	0.010 (3)	0.018 (3)	0.023 (3)	−0.003 (2)	0.000 (2)	−0.004 (2)
O4	0.016 (3)	0.015 (3)	0.011 (3)	0.001 (2)	−0.002 (2)	0.000 (2)
O5	0.014 (3)	0.020 (3)	0.011 (3)	0.004 (2)	0.002 (2)	−0.003 (2)
O6	0.019 (3)	0.011 (3)	0.019 (3)	0.003 (2)	−0.002 (2)	0.004 (2)
O7	0.018 (3)	0.020 (3)	0.019 (3)	0.003 (2)	0.008 (2)	−0.010 (2)
O8	0.008 (3)	0.016 (3)	0.013 (3)	−0.004 (2)	0.004 (2)	0.004 (2)
O9	0.017 (3)	0.019 (3)	0.015 (3)	0.003 (2)	0.010 (2)	0.007 (2)
O10	0.011 (3)	0.015 (3)	0.011 (3)	−0.002 (2)	0.005 (2)	0.003 (2)
O11	0.012 (3)	0.015 (3)	0.014 (3)	0.003 (2)	0.000 (2)	0.002 (2)
O12	0.010 (2)	0.019 (3)	0.008 (2)	0.005 (2)	0.002 (2)	0.000 (2)

### Geometric parameters (Å, °)

**Table:**

Y—O2^i^	2.282 (6)	P1—O1	1.479 (6)
Y—O5^ii^	2.296 (6)	P1—O2	1.480 (6)
Y—O9^iii^	2.358 (6)	P1—O3	1.585 (6)
Y—O11	2.363 (5)	P1—O4	1.599 (5)
Y—O12^iv^	2.387 (5)	P2—O5	1.488 (6)
Y—O6^v^	2.400 (6)	P2—O6	1.492 (6)
Y—O8	2.408 (6)	P2—O3	1.597 (6)
Y—O1	2.415 (6)	P2—O7	1.597 (6)
Y—K^i^	3.921 (2)	P3—O8	1.474 (6)
K—O12^vi^	2.736 (6)	P3—O9	1.478 (6)
K—O8^vi^	2.744 (6)	P3—O7	1.581 (6)
K—O6^ii^	2.785 (6)	P3—O10	1.594 (6)
K—O1	2.852 (6)	P4—O11	1.472 (6)
K—O9^vi^	2.859 (7)	P4—O12	1.480 (6)
K—O11^vii^	2.892 (7)	P4—O10^viii^	1.590 (6)
K—O2	2.979 (6)	P4—O4	1.598 (6)
K—O5	3.194 (6)		
			
O2^i^—Y—O5^ii^	99.8 (2)	O9^vi^—K—O11^vii^	86.49 (17)
O2^i^—Y—O9^iii^	148.9 (2)	O12^vi^—K—O2	66.57 (16)
O5^ii^—Y—O9^iii^	86.2 (2)	O8^vi^—K—O2	146.32 (18)
O2^i^—Y—O11	80.1 (2)	O6^ii^—K—O2	121.50 (18)
O5^ii^—Y—O11	147.3 (2)	O1—K—O2	50.68 (17)
O9^iii^—Y—O11	110.8 (2)	O9^vi^—K—O2	138.0 (2)
O2^i^—Y—O12^iv^	84.6 (2)	O11^vii^—K—O2	61.22 (17)
O5^ii^—Y—O12^iv^	144.7 (2)	O12^vi^—K—O5	136.07 (19)
O9^iii^—Y—O12^iv^	73.79 (19)	O8^vi^—K—O5	116.25 (17)
O11—Y—O12^iv^	67.98 (19)	O6^ii^—K—O5	54.12 (17)
O2^i^—Y—O6^v^	79.1 (2)	O1—K—O5	72.96 (18)
O5^ii^—Y—O6^v^	71.5 (2)	O9^vi^—K—O5	63.10 (17)
O9^iii^—Y—O6^v^	74.0 (2)	O11^vii^—K—O5	81.22 (17)
O11—Y—O6^v^	138.9 (2)	O2—K—O5	84.71 (17)
O12^iv^—Y—O6^v^	75.09 (19)	O1—P1—O2	115.3 (4)
O2^i^—Y—O8	140.1 (2)	O1—P1—O3	111.2 (3)
O5^ii^—Y—O8	85.16 (19)	O2—P1—O3	108.5 (4)
O9^iii^—Y—O8	70.5 (2)	O1—P1—O4	113.4 (3)
O11—Y—O8	75.39 (19)	O2—P1—O4	109.9 (3)
O12^iv^—Y—O8	113.77 (19)	O3—P1—O4	97.0 (3)
O6^v^—Y—O8	138.4 (2)	O5—P2—O6	120.0 (3)
O2^i^—Y—O1	73.9 (2)	O5—P2—O3	109.5 (3)
O5^ii^—Y—O1	75.7 (2)	O6—P2—O3	106.4 (3)
O9^iii^—Y—O1	136.7 (2)	O5—P2—O7	104.9 (3)
O11—Y—O1	72.9 (2)	O6—P2—O7	108.9 (4)
O12^iv^—Y—O1	137.92 (19)	O3—P2—O7	106.4 (3)
O6^v^—Y—O1	132.6 (2)	O8—P3—O9	116.4 (4)
O8—Y—O1	69.1 (2)	O8—P3—O7	110.1 (3)
O12^vi^—K—O8^vi^	80.69 (17)	O9—P3—O7	110.9 (4)
O12^vi^—K—O6^ii^	169.5 (2)	O8—P3—O10	107.9 (3)
O8^vi^—K—O6^ii^	91.99 (17)	O9—P3—O10	110.2 (3)
O12^vi^—K—O1	107.81 (17)	O7—P3—O10	100.1 (3)
O8^vi^—K—O1	156.9 (2)	O11—P4—O12	120.0 (3)
O6^ii^—K—O1	76.31 (17)	O11—P4—O10^viii^	107.0 (3)
O12^vi^—K—O9^vi^	118.63 (18)	O12—P4—O10^viii^	109.8 (3)
O8^vi^—K—O9^vi^	53.15 (16)	O11—P4—O4	109.3 (3)
O6^ii^—K—O9^vi^	60.95 (18)	O12—P4—O4	107.7 (3)
O1—K—O9^vi^	130.90 (19)	O10^viii^—P4—O4	101.5 (3)
O12^vi^—K—O11^vii^	56.23 (17)	P1—O3—P2	139.3 (4)
O8^vi^—K—O11^vii^	94.54 (19)	P4—O4—P1	129.2 (4)
O6^ii^—K—O11^vii^	132.5 (2)	P3—O7—P2	147.4 (4)
O1—K—O11^vii^	108.06 (18)	P4^ix^—O10—P3	130.9 (4)

Symmetry codes: (i) −*x*+1, *y*+1/2, −*z*; (ii) −*x*, *y*+1/2, −*z*; (iii) −*x*, *y*+1/2, −*z*+1; (iv) −*x*+1, *y*+1/2, −*z*+1; (v) *x*, *y*+1, *z*; (vi) *x*, *y*, *z*−1; (vii) −*x*+1, *y*−1/2, −*z*; (viii) *x*+1, *y*, *z*; (ix) *x*−1, *y*, *z*.
